# Lipid control and use of lipid-regulating drugs for prevention of cardiovascular events in Chinese type 2 diabetic patients: a prospective cohort study

**DOI:** 10.1186/1475-2840-9-77

**Published:** 2010-11-22

**Authors:** Rose ZW Ting, Xilin Yang, Linda WL Yu, Andrea OY Luk, Alice PS Kong, Peter CY Tong, Wing-Yee So, Juliana CN Chan, Ronald CW Ma

**Affiliations:** 1 Department of Medicine and Therapeutics, The Chinese University of Hong Kong, The Prince of Wales Hospital, Shatin, New Territories, Hong Kong SAR, China; 2 Li Ka Shing Institute of Health and Sciences, The Chinese University of Hong Kong, The Prince of Wales Hospital, Shatin, New Territories, Hong Kong SAR, China; 3 Hong Kong Institute of Diabetes and Obesity, The Chinese University of Hong Kong, The Prince of Wales Hospital, Shatin, New Territories, Hong Kong SAR, China

## Abstract

**Background:**

Dyslipidaemia is an important but modifiable risk factor of cardiovascular disease (CVD) in type 2 diabetes. Yet, the effectiveness of lipid regulating drugs in Asians is lacking. We examined the effects of lipid control and treatment with lipid regulating drugs on new onset of CVD in Chinese type 2 diabetic patients.

**Methods:**

In this prospective cohort consisting of 4521 type 2 diabetic patients without history of CVD and naïve for lipid regulating treatment recruited consecutively from 1996 to 2005, 371 developed CVD after a median follow-up of 4.9 years. We used Cox proportional hazard regression to obtain the hazard ratios (HR) of lipids and use of lipid regulating drugs for risk of CVD.

**Results:**

The multivariate-adjusted HR (95% confidence interval) of CVD in patients with high LDL-cholesterol (≥ 3.0 mmol/L) was 1.36 (1.08 - 1.71), compared with lower values. Using the whole range value of HDL-cholesterol, the risk of CVD was reduced by 41% with every 1 mmol/L increase in HDL-cholesterol. Plasma triglyceride did not predict CVD. Statins use was associated with lower CVD risk [HR = 0.66 (0.50 - 0.88)]. In sub-cohort analysis, statins use was associated with a HR of 0.60 (0.44 - 0.82) in patients with high LDL-cholesterol (≥ 3.0 mmol/L) and 0.49 (0.28 - 0.88) in patients with low HDL-cholesterol. In patients with LDL-cholesterol < 3.0 mmol/L, use of fibrate was associated with HR of 0.34 (0.12 - 1.00). Only statins were effective in reducing incident CVD in patients with metabolic syndrome [(HR = 0.58(0.42--0.80)].

**Conclusions:**

In Chinese type 2 diabetic patients, high LDL-cholesterol and low HDL-cholesterol predicted incident CVD. Overall, patients treated with statins had 40-50% risk reduction in CVD compared to non-users.

## Background

In this global epidemic of type 2 diabetes, Asia will have the highest prevalence with a disproportionate disease burden in young to middle aged adults [1]. Cardiovascular disease (CVD) is a leading cause of morbidity and mortality in diabetes patients resulting in poor quality of life and loss of societal productivity [2]. Controlling blood pressure, blood glucose and blood lipids reduce CVD risk in type 2 diabetic patients, including Chinese [3].

However, to date, most of the randomized clinical trials and meta-analyses were conducted in Caucasian populations with scarcity of data in Asians. In addition, a major chasm between guidelines and practice exists in developing countries, leading to delayed treatment of risk factors and preventable complications. For example, in a multinational survey involving more than 9000 type 2 diabetic patients in developing regions, 35% of patients not treated with lipid regulating drug had low-density lipoprotein cholesterol (LDL-C) ≥ 2.6 mmol/L [4]. In areas such as Hong Kong where health care system is heavily subsidized by government, prescription of a lipid-regulating drug to a diabetic patient in public clinical setting is often restricted for secondary prevention of CVD. In light of the clinical inertia, there is a need to seek evidence in Asian diabetic patients to motivate physicians to prescribe these drugs and support their use in patients without history of CVD.

In this analysis of 4521 type 2 diabetic patients, we examined the clinical utility of internationally recommended treatment targets for lipids and effectiveness of lipid regulating drugs in preventing incident CVD in Chinese type 2 diabetic patients. We also identified high-risk patients in whom the lipid regulating drugs were particularly effective.

## Methods

The Hong Kong Diabetes Registry was established in 1995 at the Prince of Wales Hospital as part of a quality improvement program. This comprehensive registry has enabled us to examine the epidemiology and impact of treatments on clinical outcomes in real practice in Chinese diabetic patients. Hong Kong is a cosmopolitan city with 7 million people, the majority of whom are of southern Chinese ethnicity. The Prince of Wales Hospital is the teaching hospital of the Chinese University of Hong Kong and serves more than 1.2 million people in her catchment area. Since 1995 and on a weekly basis, we examined 30 - 50 diabetic patients referred from community-based primary care and hospital-based specialist clinics in an ambulatory setting. The 4-hour comprehensive assessment was performed according to a protocol modified from the European DiabCare protocol [5]. Once patients underwent the assessment, their outcomes and clinical data are monitored until time of death. Written informed consent was obtained from all patients for research and publication purpose.

Between 1996 and 2005, 7387 diabetic patients were consecutively recruited in the Hong Kong Diabetes Registry. We excluded 2866 patients from this study due to having history of CVD at baseline (n = 1166), use of lipid-regulating drug at or before enrolment (n = 907), missing data for any variables included in the analysis (n = 522), having non-type 2 diabetes (n = 271). As a result, 4521 patients with type 2 diabetes without prior history of CVD and naïve to lipid-regulating drugs were included in the analysis.

All-cause death on or before July 2005 was recorded or otherwise censored on 30^th ^July 2005. We retrieved all discharge diagnoses, causes of death and drug dispensing data using the Hospital Authority Central Computer System which was used by all public hospitals in Hong Kong. A cardiovascular event was defined as coronary heart disease and/or stroke according to the International Classification of Disease, Ninth Revision [3]. Coronary heart disease was defined as the first incidence of acute myocardial infarction (code 410) and coronary death (codes 410, 411-414, and 428), nonfatal ischemic heart disease (codes 411-414), nonfatal heart failure (code 428), and coronary revascularization (procedure code 36) and percutaneous transluminal coronary angioplasty or coronary atherectomy (procedure code 00.66). Stroke was defined as first incidence of stroke (codes 430 - 434 and 436) or death from stroke (codes 430 - 438) [3]. The above data was matched by the Hong Kong identity card number, which is a unique identification number issued by the government for all the Hong Kong residents.

### Clinical and laboratory measurement

The assessment methods, definitions and laboratory assays have been described [3]. In brief, patients attended the centre after an overnight fast of at least eight hours for clinical measurements and laboratory tests. Full lipid profile consisting of total cholesterol, triglycerides and high-density lipoprotein cholesterol (HDL-C) was measured by enzymatic methods on a Hitachi 911 automated analyzer (Boehringer Mannheim, Mannheim, Germany) using reagent kits supplied by the manufacturer. The low-density lipoprotein cholesterol (LDL-C) was calculated using the Friedewald equation. All laboratory measurements were conducted by the Department of Chemical Pathology of the Prince of Wales Hospital, which was accredited by the Australian Quality Assurance Program.

### Statistical analysis

The SAS (release 9.10; SAS Institute, Cary, NC) program was used for all analysis. Follow-up time in years was estimated from enrolment to the earliest date of a CVD event, death or censoring whichever came first. We used yes/no coding for use of statins, fibrates and other drugs during the follow-up period. Cox proportional hazard regression was used to obtain hazard ratio (HR) with 95% confidence intervals (95% CI) of lipids (LDL-C, HDL-C, triglyceride) and lipid regulating drugs (i.e. statins and fibrates) for risk of CVD.

Firstly, the linearity of lipids for CVD risk was examined using restricted cubic spline Cox model analysis [6]. Restricted cubic spline consists of piecewise cubic polynomials that are connected across different intervals of a continuous variable. It can fit sharply curving shapes [6]. As in our previous analyses [7,8], we chose 4 knots at quartiles 0.05, 0.35, 0.65 and 0.95 which were suggested to offer adequate fit of the model with good compromise between flexibility and loss of precision due to overfitting of a small sample [6]. Initially, spline terms of LDL-C, HDL-C and triglyceride were entered the spline Cox model. We then performed a forward stepwise algorithm with P = 0.10 for inclusion and removal to identify possible confounding factors including clinical and biochemical covariates at enrolment, drug use during follow-up period (See table 1 for a list of candidate covariates) and years of enrolment. Based on the HR curves of lipids, we identified threshold value for CVD risk and categorized patients using these cutoff values. Otherwise, we treated the covariates as linearly associated with CVD. We performed Cox model analysis to obtain the HR of lipids for CVD with adjustments for other lipid parameters and covariates.

**Table 1:** Baseline clinical and biochemical characteristics of Chinese type 2 diabetic patients with no history of cardiovascular disease (CVD) divided according to the development of CVD during 4.9 (2.8-7.0) years of follow-up

	Non-CVD (n = 4150)		CVD (n = 371)		
	
	median or%	IQR*	median or%	IQR*	P value
Age (years)	54	21	64	16	<.0001‡
Male Gender	45.7% (1898)		52.8% (196)		.0086
Smoking status					<.0001†
Ex smoker	13.3% (553)		20.2% (75)		
Current smoker	14.9% (620)		21.2% (78)		
Waist circumference (men, cm)	87.5	12.5	88.0	11.0	.2384
Waist circumference (women, cm)	82.5	13.0	84.5	13.0	.0165
Body mass index (kg/m^2^)	24.6	5.0	24.9	4.3	.4409
Duration of diabetes (Years)	5	9	9	10	<.0001‡
Systolic blood pressure (mmHg)	132	25	141	25	<.0001‡
Diastolic blood pressure (mmHg)	75	14	75	13	.1313‡
HbA_1c _(%)	7.2	2.1	7.7	2.6	<.0001‡
Spot urine albumin creatinine ratio (mg/mmol)	1.55	5.75	7.13	54.8	<.0001‡
eGFR (ml min^-1 ^1.73 m^-2^) ξ	109	39	93	41	<.0001‡
LDL-C (mmol/L)	3.1	1.2	3.4	1.3	<.0001‡
≥3.0 mmol/L	56.1%(2330)		69.0%(256)		<.0001†
HDL-C (mmol/L)	1.28	0.45	1.18	0.45	<.0001‡
HDL-C <1.0 in male or 1.3 in female	23.8%(987)		25.9%(96)		.3655†
Triglyceride (mmol/L)	1.27	0.97	1.40	1.01	.0031‡
**Drug use at enrolment**					
Use of antihypertensive drugs at enrolment	29.7%(1231)		42.3%(157)		<.0001†
**Drug use during follow-up**					
Use of statins during follow-up	22.7%(942)		22.6%(84)		.9799†
Use of fibrates during follow-up	6.41%(266)		4.58%(17)		.1639†
Use of other lipid lowering drugs during follow-up	0.29% (12)		0.27% (1)		.3832 ††
Use of RAS inhibitors during follow-up	48.9% (2029)		62.5% (232)		<.0001†
Use of gliclazide during follow-up	43.7% (1814)		37.2% (138)		.0152†
Use of rosiglitazone during follow-up	4.9% (204)		1.6% (6)		.0038†
Use of other oral antidiabetic drugs during follow-up	79.0% (3280)		80.3% (298)		.5587†
Use of insulin during follow-up	32.4%(1345)		45.0%(167)		<.0001†
**Events during follow-up**					
Death during follow-up	5.42% (225)		27.5%(102)		<.0001†

*, Median (IQR = interquartile range); †, derived from Chi-square test; ††, Derived from Fisher's exact test; ‡, derived from Wilcoxon two-sample test; ξ, eGFR = glomerular filtration rate from modified MDRD formula; ¶, including drug use at enrolment except for statins and fibrates (users at enrolment were excluded)

We then examined the HRs of use of statins and fibrates for CVD defined as drug use from enrolment to the first CVD event, death or censoring date whichever came first. We used logistic regression procedures and a stepwise algorithm with P = 0.30 for entry and stay to select baseline covariates including age, sex, year of enrolment, waist circumference, LDL-C, HDL-C, triglyceride, smoking status, body mass index, HbA_1c_, systolic blood pressure, Ln [(urine albumin-creatinine ratio (ACR)) + 1], estimated glomerular filtration rate (eGFR), duration of disease, retinopathy and neuropathy to estimate propensity scores for adjustment of probabilities of use of statins and fibrates [9]. Age, waist circumference, LDL-C, HDL-C, triglyceride, HbA_1c_, Ln(ACR + 1), eGFR, duration of disease, retinopathy and neuropathy were selected for the propensity score for statins (c-statistic = 0.79). Age, LDL-C, HDL-C, triglyceride, HbA_1c, _systolic blood pressure, duration of disease, retinopathy and neuropathy were selected to predict use of fibrates during follow-up period (c-statistics = 0.79). Stratified models on deciles of both propensity scores were used to adjust for likelihood to use statins and fibrates during follow-up period. We further adjusted for covariates identified in the initial restricted cubic spline models to analyse the effects of statins and fibrates on CVD.

Correlations between pairs of baseline covariates were checked using Pearson's correlation test and none of the pairs were highly correlated (correlation coefficient < 0.60). Proportional hazards were checked as before. A two-sided *P *value < .05 was considered to be significant.

## Results

### Baseline characteristics

During a median follow up period of 4.9 years (interquartile range 2.8 - 7.0 years), 371 (8.2%) of 4521 subjects developed CVD. Patients who had new onset of CVD were older, more likely to be men and smokers, had longer disease duration, higher LDL-C, triglyceride, HbA_1c_, ACR, lower HDL-C and eGFR than those without. These patients were also more likely to receive insulin and less likely to receive rosiglitazone and gliclazide (Table 1). In the whole cohort, only 23% and 6% of patients were prescribed statins or fibrates respectively during follow-up.

### Lipid profile and risk for CVD

**Figure 1:**
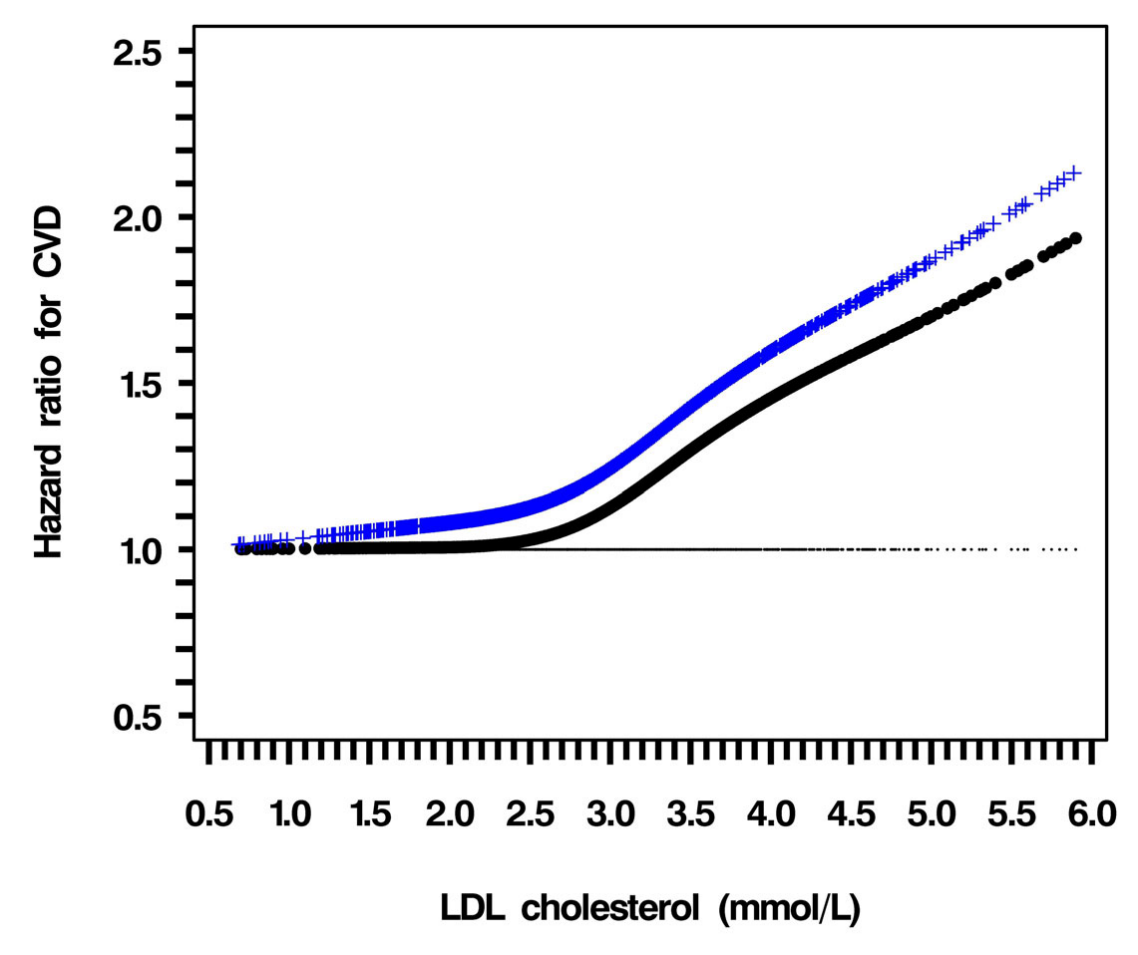
**Hazard ratio of LDL-C for risk of CVD using restricted cubic spline Cox model analysis**. Dotted curve (black), not adjusted for other covariates except for HDL-C and triglyceride; Crossed curve (Blue), Adjusted for age, smoking status, duration of diabetes, HbA1c, Ln (ACR + 1), gliclazide and rosiglitazone during follow up, years of enrolment (selected by the stepwise algorithm with P = .10 for inclusion and removal)

**Figure 2:**
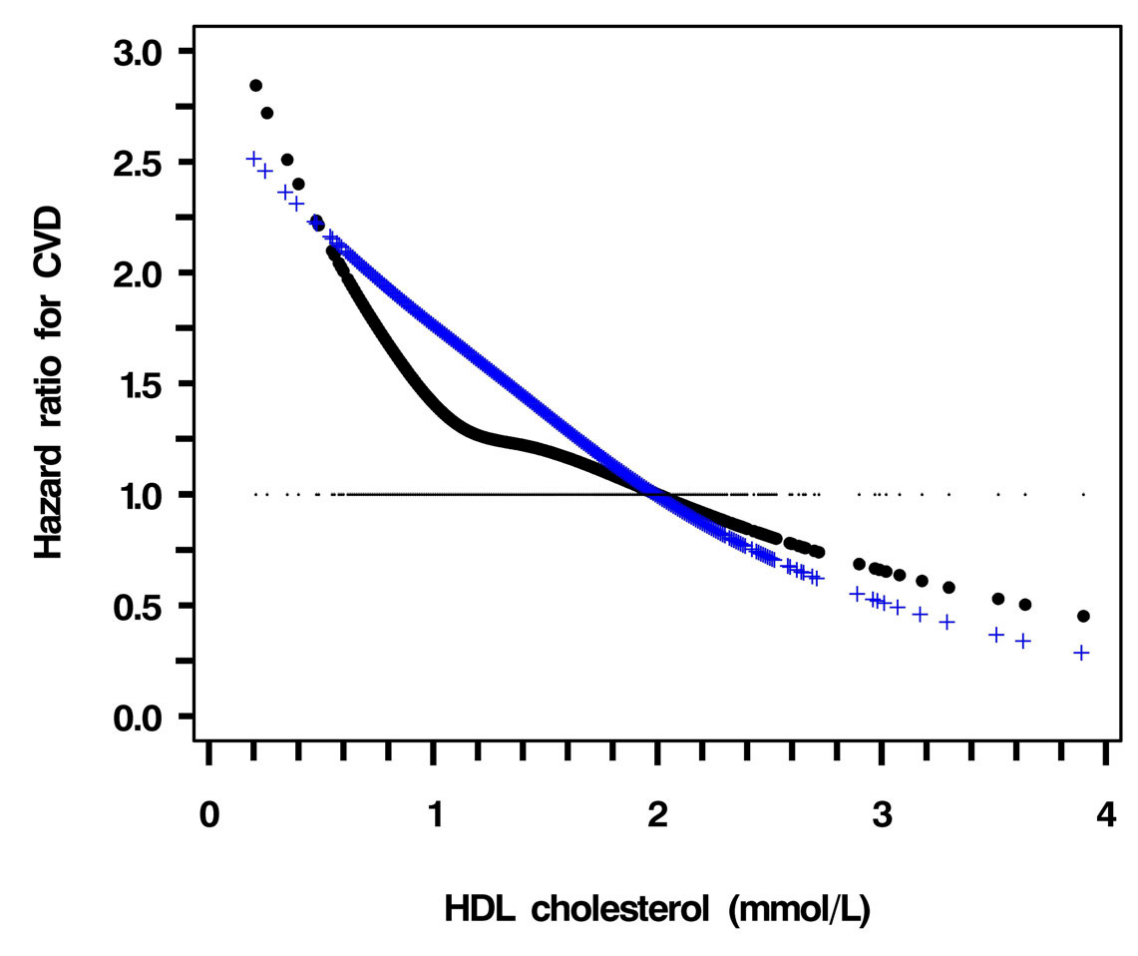
**Hazard ratio of HDL-C for risk of CVD using restricted cubic spline Cox model analysis**. Dotted curve (black), not adjusted for other covariates except for LDL-C and triglyceride; Crossed curve (Blue), Adjusted for age, smoking status, duration of diabetes, HbA1c, Ln (ACR + 1), gliclazide and rosiglitazone during follow-up, years of enrolment (selected by the stepwise algorithm with P = .10 for inclusion and removal)

**Figure 3:**
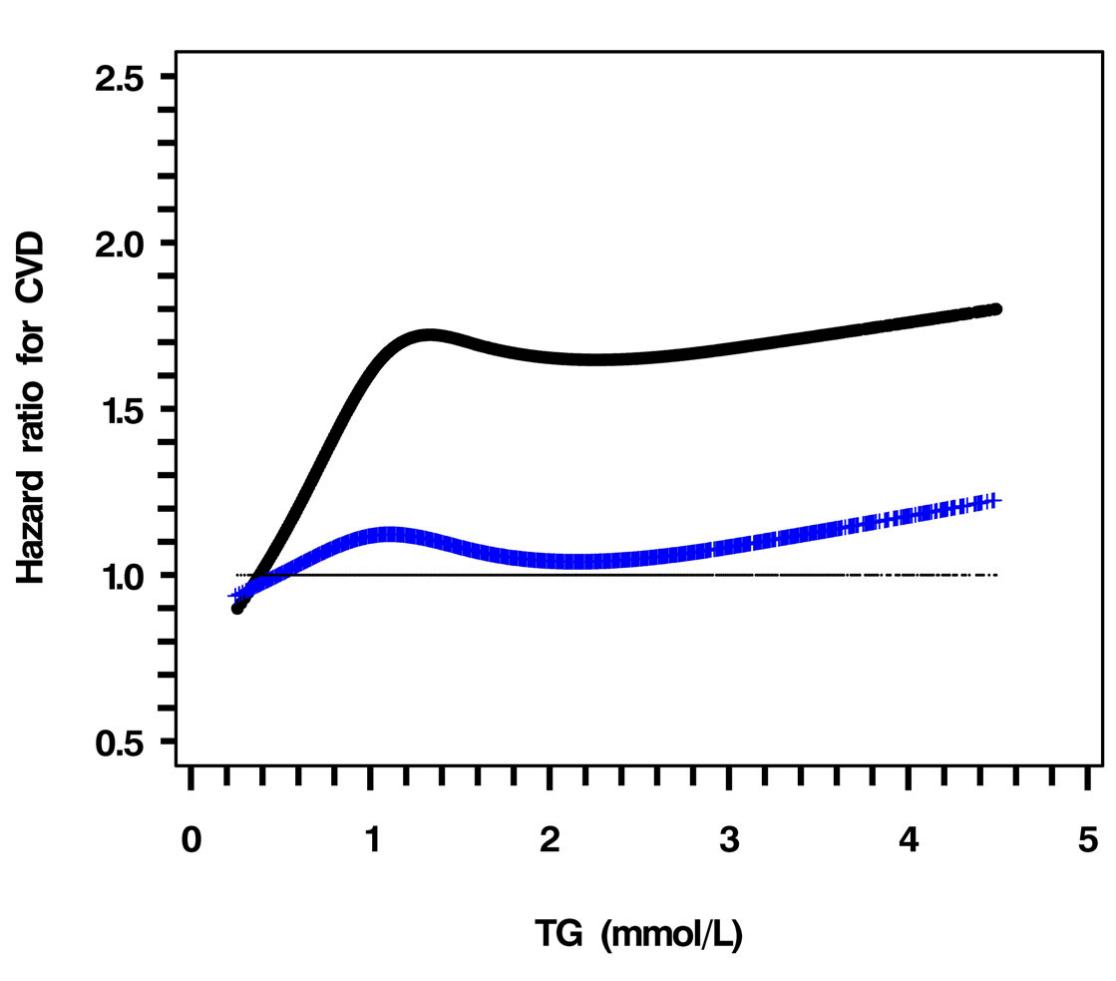
**Hazard ratio of triglyceride for risk of CVD using restricted cubic spline Cox model analysis**. Dotted curve (black), not adjusted for other covariates except for LDL-C and HDL-C; Crossed curve (Blue), Adjusted for age, smoking status, duration of diabetes, HbA1c, Ln (ACR + 1), gliclazide and rosiglitazone during follow-up, years of enrolment (selected by the stepwise algorithm with P = .10 for inclusion and removal)

In the entire cohort and before adjustment, CVD risk was positively associated with LDL-C and triglyceride but negatively with HDL-C (figure 1, black line). After adjustment for age, smoking status, duration of diabetes, HbA_1c_, Ln (ACR + 1), use of gliclazide or rosiglitazone during follow-up, years of enrolment (selected by the stepwise algorithm with P = 0.10 for entry and stay); the HR of CVD increased sharply when LDL-C was ≥ 3.0 mmol/L (Figure 1, blue line). The CVD risk declined linearly with increasing HDL-C with no definite threshold (Figure 2, blue line). The HR of CVD with triglyceride became insignificant after adjusting for confounders (Figure 3, blue line).

Using LDL ≥ 3.0 mmol/L as the cutoff point, the HR for CVD risk was 1.41 (1.13 - 1.75, *p *= 0.0024) as compared to those with LDL < 3.0 mmol/L. After adjusting for covariates, the HR was attenuated to 1.36 (1.08 - 1.71, *p *= 0.0095). Using the whole range of HDL-C values, with every 1 mmol/L increase, the HR for CVD was 0.61 (0.45 - 0.84, *p *= 0.0080), which decreased to 0.59 (0.42 - 0.82, *p *= 0.0020) after adjustment for covariates (Table 2).

**Table 2:** Hazard ratios of lipid profile for risk of cardiovascular disease in Chinese type 2 diabetic patients

	HR	95% CI	P value
Model One†			
LDL-C ≥ 3.0 mmol/L vs. <3.0 mmol/L	1.41	1.13 to1.75	.0024
HDL-C, mmol/L	0.61	0.45 to 0.84	.0080
Triglyceride, mmol/L	1.08	0.97 to 1.21	.1669
Model Two‡			
LDL-C ≥ 3.0 mmol/L vs. <3.0 mmol/L	1.36	1.08 to 1.71	.0095
HDL-C, mmol/L	0.59	0.42 to 0.82	.0020
Triglyceride, mmol/L	1.02	0.88 to 1.17	.9614

†, Not adjusted for other covariates;

‡, Adjusted for age, smoking status, duration of diabetes, HbA1c, Ln (ACR + 1), use of statins, fibrates, gliclazide and rosiglitazone during follow-up, years of enrolment (selected by the stepwise algorithm with P = .10 for entry and stay).

### Use of lipid lowering drugs and risk for CVD

In this new-user cohort, use of statins reduced CVD risk by 34% after controlling for baseline covariates and use of other medications during follow-up. Use of fibrates did not reduce CVD risk after adjustment for other covariates (Table 3). The trend of risk reduction was consistent in nearly all subgroups stratified by LDL-C and HDL-C levels, reaching significance for some subgroups. In patients with LDL-C ≥ 3 mmol/L, statins significantly reduced CVD risk by 40%. In patients with LDL-C < 3 mmol/L, fibrates reduced CVD risk by 66%. In patients with low HDL-C (< 1.0 mmol/L in men and < 1.3 mmol/L in women) and those with concomitant metabolic syndrome [10], statins reduced CVD risk by 51% and 42% respectively.

**Table 3:** Hazard ratios of use of statin and fibrate for risk of cardiovascular disease in Chinese type 2 diabetic patients

Exposures	HR	95%CI	P value
**In the entire cohort**§†			
Use of statins during follow-up	0.66	0.50 to 0.88	.0038
Use of fibrates during follow-up	0.61	0.37 to 1.03	.0640

**In the sub-cohort with LDL-C < 3.0 mmol/L¶, §**			
Use of statins during follow-up	1.10	0.56 to 2.16	.7798
Use of fibrates during follow-up	0.34	0.12 to 1.00	.0495
**In the sub-cohort with LDL-C ≥ 3.0 mmol/L¶, §**			
Use of statins during follow-up	0.60	0.44 to 0.82	.0012
Use of fibrates during follow-up	0.65	0.35 to 1.19	.1590

**In the sub-cohort with HDL-C < 1.0 mmol/L in male or 1.3 mmol/L in female ζ, §**			
Use of statins during follow-up	0.49	0.28 to 0.88	.0162
Use of fibrates during follow-up	0.37	0.13 to 1.09	.0703
I**n the sub-cohort with HDL-C ≥1.0 mmol/L in male or ≥ 1.3 mmol/L in female ζ, §**			
Use of statins during follow-up	0.73	0.53 to 1.02	.0662
Use of fibrates during follow-up	0.74	0.41 to 1.35	.3303

I**n the sub-cohort with metabolic syndrome at baseline ‡,§**			
Use of statins during follow-up	0.58	0.42 to 0.80	.0010
Use of fibrates during follow-up	0.60	0.35 to 1.05	.0713
I**n the sub-cohort without metabolic syndrome at baseline ‡,§**			
Use of statins during follow-up	0.77	0.42 to 1.44	.4201
Use of fibrates during follow-up	0.57	0.12 to 2.73	.4903

†Adjusted for LDL-C (≥ 3.0 mmol/L vs. < 3.0 mmol/L), HDL-C, triglyceride, age, smoking status, duration of diabetes, HbA1c, Ln (ACR + 1), gliclazide and rosiglitazone during follow-up, years of enrolment (selected by the stepwise algorithm with P = .10 for entry and stay);

Adjusted for the variables listed in ‡, except for LDL-C;

ζ, Adjusted for the variables listed in ‡, except for HDL-C;

§, Cox models stratified on deciles of probability of initiation of statin therapy during follow-up and on deciles of probability of initiation of fibrate therapy during follow-up.

## Discussion

Our study results revealed high LDL-C and low HDL-C, but not triglyceride, predicted incident CVD in Chinese type 2 diabetic patients. Using spline analysis, the optimal LDL-C level for CVD risk was <3 mmol/L while HDL-C exhibited a continuous and linear relationship with CVD risk. Use of statins was associated with 34% risk reduction in CVD while the risk reducing effect of fibrates was rendered insignificant after adjusting for confounders. On subgroup analysis, statins use reduced CVD risk by 40% in patients with LDL-C≧3.0 mmol/L and by 51% in those with low HDL-C (<1.0 mmol/L in male and <1.3 mmol/L in female) and 42% in those with metabolic syndrome. In patients with LDL-C<3 mmol/L, fibrates use was associated with 66% risk reduction of CVD with borderline significance.

### Lipid profile, use of lipid regulating drugs and CVD risk

Epidemiological studies and interventional trials confirmed the predictive value of high LDL-C on risk of CVD. These risks were accentuated by 2-3 folds in type 2 diabetic patients for the same number of risk factors [11,12]. Furthermore, type 2 diabetic patients are often insulin resistant with a typical dyslipidaemic pattern of low HDL-C and high triglyceride [13-15]. Despite the similar LDL-C levels between type 2 diabetic and non-diabetic subjects, the high triglyceride contributes to increased concentration of small dense LDL-C particles which are more atherogenic. Type 2 diabetic patients are also more likely to have oxidized and glycated LDL-C which are readily taken up by macrophages to form atheromatous plaques. Given the consistency of interventional data, lowering of LDL-C using HMGcoA reductase inhibitors (statins) remains the primary target in preventing CVD in type 2 diabetes [2,16,17].

As early as in 1977, the Framingham study demonstrated an inverse association between HDL-C and incidence of coronary heart disease after adjustment for other lipid parameters and risk factors [18]. In the Asia Pacific Cohort Studies Collaboration consisting of more than 50,000 data, HDL-C, total cholesterol and LDL-C were independent risk factors for coronary heart disease in Asians [19]. By contrast, the prognostic significance of triglyceride in CVD remains controversial [19-23]. While some researchers have reported triglyceride to be the most predictive lipid parameter for CVD, in a recent meta-analysis by the Emerging Risk Factors Collaboration, such risk association was not confirmed after controlling for other confounders [23]. In this analysis, triglyceride also did not predict incident CVD in our Chinese type 2 diabetic patients after adjusting for confounders.

In Caucasians, meta-analyses of large scale randomized studies [15,24,25] have confirmed the benefits of statins on CVD in primary and secondary prevention studies which included diabetic and non-diabetic subjects. These beneficial effects have recently been extended to patients with LDL-C level less than the recommended treatment level of 2.6 mmol/L although these patients had high sensitivity C reactive protein [26]. In the post-hoc analysis of the FIELD study, treatment with fenofibrate reduced the risk of CVD by 27% in a subgroup of type 2 diabetic patients with high triglyceride and low HDL-C [27]. Echoing this finding, the recently published ACCORD lipid study showed benefit of fenofibrate in a subgroup of type 2 diabetic patients who had both high triglyceride ≥ 2.30 mmol/L and low HDL-C ≤ 0.88 mmol/L [28]. We found use of statins was associated with reduced risk of CVD in the whole group as well as in those with high LDL-C, low HDL-C and metabolic syndrome. The benefit of fibrates on CVD in the whole group was rendered insignificant after adjusting for confounders, probably due to the small sample size.

Recent genetic studies suggested possible inter-ethnic differences in patterns of risk variants associated with dyslipidaemia. For example, two single nucleotide polymorphisms of the KCNQ1 gene, a well-known type 2 diabetes gene, were associated with higher level of triglycerides and lower levels of HDL-C in Chinese population [29]. On the other hand, in Chinese type 2 diabetic patients with concomitant coronary heart disease, the PPARγC161 ༠ T genotype was associated with reduced severity of atherosclerosis, in part medicated by improved lipid metabolism of decreased the triglycerides and apoB levels [30]. While these findings suggest that triglyceride may be implicated in CVD in Chinese subjects, after adjusting for cofounders, our overall results concord with most studies regarding the importance of LDL-C and statins in modifying CVD risk in Chinese.

### Overcoming bias in pharmacoepidemiological study

Despite the growing epidemic of diabetes and expected increase in CVD in Asia, there have not been any large scale clinical trials to examine the efficacy of use of statins or fibrates in preventing CVD in Asian populations. In the absence of such evidence, treatment recommendations were largely empirical and derived from Western guidelines. To our knowledge, our present analysis is by far the largest prospective cohort of Asian type 2 diabetic patients with detailed documentation of risk factors, drug usage and clinical outcomes. Our diabetes registry provided useful information for guiding clinical practice and for future large randomized clinical trials in Chinese.

To circumvent potential bias inherent in an observational survey, we have used a new-user design and excluded existing statins or fibrates users. Such design is superior to traditional cohort studies as prevalent users cause indication and adherence bias [31]. Besides, all patients have undergone comprehensive assessment with detailed documentation of other confounders which were adjusted in our analysis. We further used propensity score to adjust for bias due to prescribers' preference although we were unable to fully adjust for factors such as volunteer bias which is often seen in patients more compliant to treatment or follow up. After careful analysis of the bias, we concluded that the drug use definition was robust and did not introduce major bias neither towards an increased nor decreased risk of the endpoint [32].

### Limitations

Our results need to be interpreted with the following caveats. We only measured a single fasting plasma lipid profile when plasma triglyceride level is known to have marked day-to-day and intra-individual variability. This may have attenuated the risk association between triglyceride level and CVD outcomes. Our cohort was predominantly clinic-based although given the heavily-subsidized health care system in Hong Kong, our public hospital clinics provide care to more than 90% patients with chronic diseases such as diabetes [7]. Despite our careful adjustment for potential confounders, a new-user cohort design cannot replace a randomized controlled trial which aims to remove bias due to unbalanced distribution of measured and unmeasured confounders.

In our survey, lipid levels were not systematically collected during clinic visits after the initial enrolment. To avoid bias due to unknown lipid levels during start of therapy, we opted to use yes/no coding rather than time-dependent use in our analysis. In these public hospital clinics, atorvastatin, fluvastatin, pravastatin, rosuvastatin and simvastatin were available with atorvastatin (10-20 mg daily) and simvastatin (10-40 mg daily) being the two most popular statins. For fibrates, bezafibrate and gemfibrozil were both available with gemfibrozil being the more popular choice. However, with the relatively small sample size of patients on statins and fibrates, we were not able to examine the effects of individual drug on CVD. We also did not document the drug compliance of the patients or check whether other private drugs were used. Although non compliance or occasional drug omission is not uncommon in patients on long term medications, this would tend to attenuate effect size and level of significance. Besides, surveys by our other colleagues have reported more than 90% compliance amongst patients taking lipid regulating drugs [33,34].

## Conclusions

In conclusion, in a relatively large prospective cohort of Chinese type 2 diabetic patients without past history of CVD, both LDL-C (≧ 3.0 mmol/L) and HDL-C level but not the triglyceride level predicted incident CVD. Use of statins was associated with reduced CVD risk in patients with high LDL-C level, low HDL-C level or metabolic syndrome while fibrates use was associated with reduced CVD in patients with low LDL-C Given the narrow risk-benefit ratio of intensive blood glucose lowering in type 2 diabetic patients especially those with long disease duration who often have silent ischemic disease [35], patients and health-care professionals must be aware of the importance of attaining recommended lipid goals to reduce CVD risks.

## Competing interests

Professor Juliana Chan has received research grants, consultancy and speakers fees from Astra-Zeneca, Bayer, Daiichi-Sankyo, MSD, Pfizer, Lilly and Sanofi-Aventis which have been donated to the Chinese University of Hong Kong to support research and education in diabetes. Professor Juliana Chan and Dr. Ronald Ma are members of an advisory board on lipids and cardiovascular risk in Asia supported by Pfizer.

## Authors' contributions

JC conceptualized the study and finalised the manuscript. RT drafted the manuscript and analysed the data. XY designed the study and analysed all data. LY collected all data and helped analysing the data. AL, AK, PT, WS, RM gave advice to the study and edited the manuscript. All authors read and approved the final manuscript.
